# Leucémie aigüe myélomonocytaire à éosinophiles révélée par une pancréatite aigüe

**DOI:** 10.11604/pamj.2015.20.112.6024

**Published:** 2015-02-09

**Authors:** Aziz Touaoussa, Khalid Elhmadi, Hind El Youssi, Hichameddou Moncef, Moncef Amrani Hassani

**Affiliations:** 1 Laboratoire Central d'Analyses Médicales, Laboratoire d'Hématologie, CHU Hassan II, Atlas Fès, Maroc; 2 Service de Laboratoire d'Hématologie, de l'Hopital My Ismail de Meknes, Maroc; 3 Service d'Hematologie, Clinique de l'Hopital My Ismail de Meknes, Maroc

**Keywords:** LAM4, pancréatite, aigue, paroxétine, Acute Myelocytic Leukemia 4, pancreatitis, acute, paroxetine

## Abstract

La leucémie aigüe myélomonocytaire à composante éosinophile (LAM4eo) est une hémopathie maligne rare, caractérisée par une prolifération blastique myéloïde avec présence d'une composante monocytaire et des éosinophiles anormaux. Elle est associée à l'inversion du chromosome 16, parfois à sa variante la translocation (16;16). Nous rapportons un cas de LAM4eo chez un patient de sexe masculin âgé de 51 ans, découverte au décours d'un bilan paraclinique pour pancréatite aiguë (PA) confirmée par la TDM. L'hémogramme a montré une hyperleucocytose à 123G/l faite de 60% de blastes, une monocytose à 5 G/L ainsi qu'une lignée éosinophile dystrophique. Le myélogramme a objectivé l'infiltration de la moelle par une population blastique estimée à 61% d'expression hétérogène à la Cytométrie de flux: Des blastes très immatures exprimant fortement les marqueurs CD117 et CD34; des blastes prédominants qui expriment les antigènes CD33, CD13, CD65 (myéloblastes). Et une partie des blastes, était positive pour le CD14, CD4, CD11c (monoblastes). Après une exploration étiologique approfondie n'ayant pas pu trouver un lien de cause à effet, l'association entre les deux pathologies a été considérée comme fortuite et la pancréatite a été rattachée à la prise de paroxétine. Le patient a été mis alors en condition, et a été traité par chimiothérapie avec bonne évolution clinique et biologique.

## Introduction

La leucémie aigue myélomonocytaire à composante éosinophile, LAM4 Eo. Selon la classification internationale Franco-Américano-Britannique (FAB), est une maladie rare, représentantentre 5 à 8% de l'ensemble des leucémies aigues myéloïdes, dont elle se distingue par son meilleur pronostic [1], avec un taux de rémission de l'ordre de 76% à 92%. Nous rapportons un cas de LAM4 Eo chez un patient de 51 ans, révélée par une pancréatite aiguë.

## Patient et observation

Il s'agit d'un patient de sexe masculin, âgé de 51 ans, de race blanche,ayant comme antécédents médicaux une hyperuricémie traitée par l'allopurinol, une dépression pour laquelle le patient a été mis sous paroxétine il y'a 15 jours, et un éthylisme sevré depuis 10 ans. 40jours avant son admission, le patient s'est plaint d'une asthénie s'aggravant progressivement et d'un amaigrissement non chiffré, sur cette symptomatologie de fond s'est greffée soudainement des épigastralgies intenses transfixiantes, accompagnées de vomissements et de fièvre. Ce qui a motivé son adresse par un médecin généraliste au service des urgences

A l'admission, l'examen clinique a montré un patient en mauvais état général, pâle, fébrile (38.5 C), avec une tachycardie à 95 battement/min, et une hypotension artérielle à 10/06mm Hg. L'examen de l'abdomen a montré une défense, avec sensibilité localisée à la région épigastrique, ainsi qu'une hépato-splénomégalie. L'examen cutanéo muqueux a montré la présence d'un purpura pétéchial, localisé au niveau des deux bras et des jambes, sans ecchymoses et sans papules (leucémides), notamment pas d'ictère. A L'examen ORL on a noté une otite moyenne aigue purulente droite. Alors que l'examen de la cavité buccale était normal: absence d'hypertrophie gingivale et amygdalienne.

**Figure 1:**
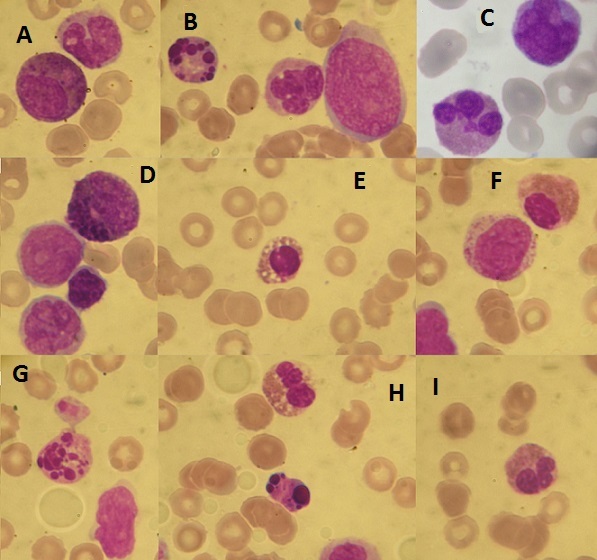
Frottis sanguin (May-Grünwald-Giemsa-objectif x 100) montrant les dystrophies de la lignée éosinophile: (A): un précurseur éosinophile anormal contenant à la fois des granules éosinophiles et de larges granules basophiles; un monocyte; (B): blastes d'allure indifférenciée; monocyte et cellule dysplasique au noyau fragmenté; (C): polynucléaire éosinophile avec un noyau hypersegmenté; (D): blaste d'allure indifférenciée, précurseuréosinophile contenant des granulations basophiles immatures et un blaste d'allure monocytaire; (E): polynucléaire éosinophile avec un noyau monolobé et un cytoplasme vacuolé; (F): polynucléaire éosinophile avec un noyau monolobé; (G): cellule dysplasique avec un noyau fragmenté; (H): polynucléaire éosinophile avec un noyau hypersegmenté, et une cellule dysplasique; (I): polynucléaire éosinophile avec un noyau hypersegmenté

**Figure 2:**
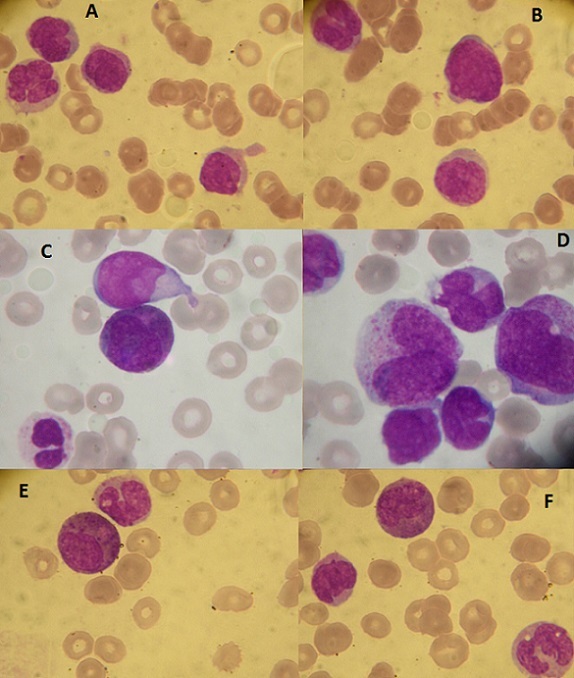
Frottis sanguin (May-Grünwald-Giemsa-objectif x 100) montrant différents types de blastes dont certains sont dystrophiques: (A) et (B): présence de blastes d'allure indifférenciée, de monocyte et de blastes dystrophiques aux noyaux irréguliers; (C): un blaste d'allure indifférenciée; un précurseur de la lignée éosinophile dystrophique contenant à la fois des granules éosinophiles normaux et de larges granules basophiles; (D): présence de myéloblastes et de monoblastes avec des noyaux irréguliers; (E) et (F): monocyte et précurseur éosinophile contenant de larges granules basophiles

L'examen des aires ganglionnaires a objectivé la présence d'adénopathies cervicales et inguinales bilatérales centimétriques fermes et indolores. Le reste de l'examen clinique était sans particularité notamment: un examen neurologique normal éliminant une éventuelle infiltration blastique neuro méningée, et un examen pleuro pulmonaire sans particularités. Un bilan biologique a été demandé et a montré: à la NFS une hyperleucocytose majeur à 123 G/L, avec une anémie normochrome normocytaire à 4g/dl, et une thrombopénie profonde à 14 G/L. Le frottis sanguin a permis de réaliser une numération qui a montré la présence d'une blastose à 60%, une monocytose à 5 G/L, et un excès de polynucléaires éosinophiles dysplasiques (noyau monolobé, trilobé, granulations immatures, vacuoles cytoplasmiques) (Figure 1 et Figure 2).

**Figure 3:**
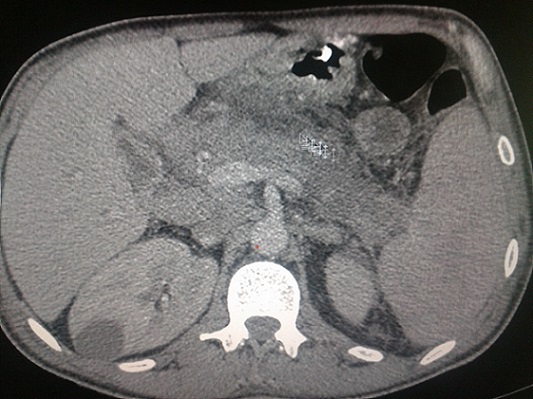
TDM abdominale avec injection de produit de contraste iodé: coupe transversal

**Figure 4:**
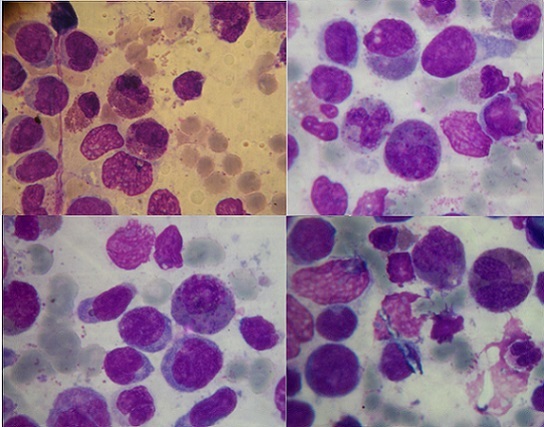
Myélogramme (coloration MGG; X 100) montrant l'infiltration de la moelle osseuse par des myéloblastes, des monoblastes et des précurseurs éosinophiles dystrophiques

Le bilan biochimique a montré les résultats suivants: une Lipasémie à 1063 UI/l, une glycémie à 1.18 g/l, un bilan hépatique normal (pas de cholestase ni cytolyse), une hypocalcémie à 73g/l,des triglycérides à 1.80 g/l, une C réactive protein (CRP) à 160mg/l, et l'acide urique à 94 mg/l. Un Scanner abdominal réalisé en urgence avec injection de produit de contraste a révélé la présence d'un pancréas augmenté de volume dans son ensemble, avec perte de l'aspect lobulé, et une coulée de nécrose péri pancréatique confirmant le diagnostic de PA stade D de Balthazar. Il a montré en outre, la présence d'une cholécystite alithiasique, et la présence de multiples adénomégalies lombo aortiques et inguinales infra centimétrique (Figure 3). Un myélogramme a été réalisé et a mis en évidence une moelle hypercellulaire, infiltrée par 61% de blastes, faits de myéloblastes,et de blastes indifférenciés d'allure monocytaire (Figure 4). La composante éosinophile tout stade de maturation confondus s'estimait à 18%, dont la majorité était dysplasique. Une activité enzymatique peroxydasique était positive à 65%. Le phénotypage par Cytométrie en flux amontré l'existence: de blastes très immatures exprimant fortement les marqueurs CD117 et CD34. Des blastes prédominants qui expriment les antigènes CD33, CD13, CD65 (myéloblastes). Et une partie des blastes, qui était positive pour le CD14, CD4, CD11c (monoblastes).

Au terme de ce bilan le diagnostic de PAet de LAM4 éosinophile ont été retenus. Au service de réanimationle patient a été mis sous nutrition parentérale avec correction des troubles hydro-électrolytiques, associés à un traitement antalgique, et une antibiothérapie à large spectre (Amikacine + Rocéphine), relayés secondairement par la Vancomycine après dépistage de *Staphylococcus aureus Méti-R* au niveau nasal. Après un bilan pré thérapeutique,une chimiothérapie d'induction a été démarrée. A j + 5, la fièvre avait disparu, et la Lipasémie a commencé à baisser progressivement. A j + 26, le patient s'est rétabli et a été inscrit sortant, avec un rendez pour bilan d’évaluation de sa réponseà la chimiothérapie, et un éventuel traitement d'entretien.

## Discussion

La LAM4 avec éosinophiles anormaux selon l'ancienne appellation de la classification FAB a été associée à l'inversion du chromosome 16 [2], laquelle fait partie selon la classification 2008 de l'OMS des leucémies aiguës myélomonocytaires avec anomalies cytogénétiques récurrentes plus particulièrement du groupe des LAM impliquant les gènes CBF (Core Binding Factor), regroupant les LAM 2 avec t(8;21) (q22;q22) et les LAM4Eo avec inv (16) ou t (16;16) (p13;q22) (p13;q22), dont la conséquence est la fusion des gènes CBF bêta localisé en 16q22 [3] et du gène *Smouth Muscle Myosin Heavy Chain (MYH11)* codant pour la chaîne lourde de la myosine, avec production de 11 transcrits différents, dont le plus fréquent est le type A [4], et qui aurait deux conséquences majeurs: d'une part une séquestration de la protéine codée par le gène AML1 (nommé aussi CBFA2 ou RUNX1) - et qui est serait impliquée dans le contrôle de la différentiation hématopoïétique [5] au niveau cytoplasmique,l'empêchant d'accéder au noyau [6]. Et d'autre part une répression de la transcription de facteurs impliqués dans l'hématopoïèse, principalement par le biais du recrutement d'histones désacétylases [7], qui bloquent l'accès des complexes de transcription à l'ADN, d'où un blocage de la différentiation [8]. Mais cette inversion ou translocation (mutation de type II), reste insuffisante pour entrainer à elle seule une leucémie,et nécessite l'association d'autres mutations (type I), en effet près de 70% des patients atteints de LAMavecinv(16) sont connus porteurs de l'une des mutationsintéressant les récepteurs des tyrosines kinases, à savoir: RTK, c-KIT etFLT3, ainsi quedes gènesRAS, qui sont responsable de l'effet prolifératif et de la survie du clone muté [8].

L'inversion du chromosome 16 est une anomalie qui reste difficilement visible au caryotype standard en bandes R, surtout si la métaphase est de mauvaise qualité [9]. La détection du réarrangement des gènes CBF bêta / MYH11 peut se faire alors par les techniques d′hybridation in situ révélée par fluorescence (FISH), et ce, quelle que soit la qualité des métaphases [10]. Son transcrit de fusion quant à lui peut être révélé par RT-PCR, permettant, en plus d'une aide diagnostique, un suivi de la maladie résiduelle [11].

Le traitement de cette hémopathie maligne à base de chimiothérapie se déroule en deux phases: Une phase d'induction intensive se déroulant sur la base de l’étalon-or, selon le schéma 3 + 7 (trois jours d'anthacycline se recoupant avec sept jours d'AraC) avec comme objectif: une rémission morphologique complète. Une phase de consolidation constituée de plusieurscycles d'AraC à haute dose [12]. Notre patient a pu bénéficier du même protocole, avec une bonne réponse clinique à savoir une rémission de sa LAM4 et une régression des signes cliniques en rapport avec sa PA. Mais demeure la question: existe-t-il un lien entre ces deux pathologies? Malheureusement la revue de la literature n'a pas été fructueuse et ne nous a pas permis de trouver un cas similaire sur lequel on puisse fonder une hypothèse. Dans les rares cas d'association entre une leucémie aigüe et une PA. Il s'agissait soit d'une leucémie aigue lymphoblastique entrainant une hypercalcémie [13] à l’ origine de la PA, ce qui n'est pas le cas de notre patient souffrant au contraire d'une hypocalcémie traduisant probablement une cyto-stéatonécrose. Soit de pancréatites iatrogènes, post chimiothérapie à base de Cytarabine, [14] ou de L-asparaginase [15]. Renonçant à l'hypothèse d'une relation de cause à effet entre la LAM4 et la PA, on a entrepris une enquête étiologique standard en debutant par les étiologies les plus fréquentes de la PA.C'est ainsi qu'on a commencé par écarter l'alcoolisme, vu le sevrage qui remonte à plus de dix ans selon le patient,et vue l'absence actuelle de signes biologiques d'imprégnation alcoolique en l'occurrence: un volume globulaire moyen augmenté, et des Gamma Glutamyl-Transpeptidase élevées.

Un bilan radiologique nous a permis d’éliminer également une origine lithiasique, consolidé par un bilan biologique qui n'a pas montré de pic des transaminases ni d’élévation de la bilirubine,reflets de la lithiase de la voie biliaire principale. Le bilan radiologique nous a permis aussi d’éliminer l'hypothèse d'une compression tumorale du canal pancréatique principal. Après quoi le patient a bénéficié d'une sérologie virale pour herpès virus (Herpesviridae) (HSV, VZV, CMV) et qui était négative. C'est alors qu'on s'est orienté vers la voie des PA post médicamenteuse, incriminant le paroxétine, responsable de quelques rares cas de PA déjà publiés [16]. Pour établir la relation d'imputabilité entre un médicament et la PA, Mallory et coll [17] ont établi quatre critères: d'abord la pancréatite doit se développer durant le traitement, et ce indépendamment du délai entre la prise du médicament et l'apparition des symptômes, puisque Biour et coll [18] ont montré que certains médicaments pouvaient engendrer une PA avec parfois des délais pouvant atteindre plusieurs mois après l′introduction de l′agent pharmacologique. Le deuxième critère est l′absence d′autres causes de PA; le troisième c'est le bon pronostic et la résolution après interruption du médicament; et enfin la pancréatite doit récidiver avec la réintroduction du médicament. L'association certaine entre le médicament et la PA requiert les quatre critères à la fois, alors qu'une association probable en requiert uniquement trois, c'est-à-dire excluant la réintroduction du médicament puisque attitude dangereuse. La survenue de la PA chez notre patient au cours du traitement par le paroxétine, l'absence d'une autre cause évidente, ainsi que l'amélioration de son état clinique et biologie après arrêt du traitement étaient les critères sur lesquels on s'est basé pour établir un probable lien entre le paroxétine et la PA.

## Conclusion

La Leucémie aigüe myélo-monocytaire à composante éosinophile (LAM4eo) est une hémopathie maligne rare, de meilleur pronostic par rapport aux autres leucémies myéloïdes, Sauf que dans notre cas elle été associée de façon fortuite à une PA, pathologie redoutée pour ses complications. Heureusement le patient a bien répondu au traitement avec bonne évolution clinique et biologique, ce qui nous réconforte dans notre hypothèse d'une PA post médicamenteuse, probablement au paroxétine.
